# Compressive Sensing-Based Radar Imaging and Subcarrier Allocation for Joint MIMO OFDM Radar and Communication System

**DOI:** 10.3390/s21072382

**Published:** 2021-03-30

**Authors:** SeongJun Hwang, Jiho Seo, Jaehyun Park, Hyungju Kim, Byung Jang Jeong

**Affiliations:** 1 Division of Smart Robot Convergence and Application Engineering, Department of Electronic Engineering, Pukyong National University, Busan 48513, Korea; never9898@naver.com (S.H.); sjs9575@naver.com (J.S.); 2 Radio & Satellite Research Division, Communication & Media Research Laboratory, Electronics and Telecommunications Research Institute, Daejeon 34129, Korea; kimhyungju@etri.re.kr (H.K.); bjjeong@etri.re.kr (B.J.J.)

**Keywords:** MIMO OFDM radar and communication, subcarrier allocation strategy, Bayesian matching pursuit

## Abstract

In this paper, a joint multiple-input multiple-output (MIMO OFDM) radar and communication (RadCom) system is proposed, in which orthogonal frequency division multiplexing (OFDM) waveforms carrying data to be transmitted to the information receiver are exploited to get high-resolution radar images at the RadCom platform. Specifically, to get two-dimensional (i.e., range and azimuth angle) radar images with high resolution, a compressive sensing-based imaging algorithm is proposed that is applicable to the signal received through multiple receive antennas. Because both the radar imaging performance (i.e., the mean square error of the radar image) and the communication performance (i.e., the achievable rate) are affected by the subcarrier allocation across multiple transmit antennas, by analyzing both radar imaging and communication performances, we also propose a subcarrier allocation strategy such that a high achievable rate is obtained without sacrificing the radar imaging performance.

## 1. Introduction

Recently, interest in autonomous driving and connected car has grown because of implementing an intelligent transportation system. Although sensors such as light detection and ranging (Lidar) and cameras can be used to recognize the surrounding environment, vehicle radar technology using millimeter waves is drawing attention as it is not affected by the surrounding environment, such as bad weather or light intensity [1,2]. In order to increase the number of sensors fitted to vehicles and to communicate various sensing information and road information, inter-vehicle communication technology with a high data rate is needed. For general vehicle radars, it is important to sense forward-looking vehicles and obstacles, but the resolution of range and azimuth angle is poor with limited frequency bandwidth and limited number of antennas. In addition, it is necessary to consider the potential interference between radar and mobile communication systems in a situation where communication traffic among vehicles increases, also being discussed in 5G mobile communication community [3].

Due to high-frequency efficiency and ease of interference management, orthogonal frequency division multiplexing (OFDM) radar has been extensively investigated [4]. In [5,6] (and references therein), symbol-based signal processing is proposed to estimate range profile without correlation based baseband signal processing, which also motivates the joint radar and communication (RadCom) systems [7,8,9]. In [8], the OFDM waveforms carrying the modulated data in each subcarrier are exploited to obtain the range-Doppler map, where the payload data is canceled out in the received OFDM baseband signal at RadCom platform. In [9], the bistatic RadCom system is developed using OFDM waveforms by exploiting linear array antennas at both transmitter and receiver, and the target parameters (i.e., range, Doppler, and azimuth angle) are estimated by using alternative least squares algorithm. To obtain a two-dimensional (2D) spatial (i.e., range and azimuth angle) radar image, OFDM radar equipped with multiple antennas has been also investigated [10]. In [10], a multiple signal classification (MUSIC) algorithm is applied to each range bin for the azimuth angle estimation. However, in the MUSIC algorithm, the estimation of covariance matrix of the received signal is required, which may incur a considerable latency to collect multiple OFDM waveforms at the multiple receive (Rx) antennas. In [11,12,13], compressed sensing-based radar imaging algorithms have been developed for multiple-input multiple-output (MIMO) OFDM radar, not requiring the subspace estimation. However, they do not consider the communication performance when multiple antennas are deployed at RadCom platform.

In this paper, by considering the RadCom platform equipped with multiple antennas using OFDM waveform (i.e., MIMO OFDM RadCom platform), we propose the compressive sensing-based radar imaging and the subcarrier allocation methods. Specifically, to get 2D radar images with high resolution, a compressive sensing-based imaging algorithm is first proposed when the subcarriers are orthogonally allocated across multiple transmit (Tx) antennas. Differently from the compressive sensing based estimation for MIMO OFDM radars in [11,12,13], where basis pursuit (BP) or orthogonal matching pursuit (OMP) algorithms are exploited, the Bayesian matching pursuit (BMP)-based imaging method (which is successfully applied to the FMCW MIMO radar system [14]) is developed for the MIMO OFDM RadCom platform. Because both the mean square error (MSE) of the radar image and the achievable rate are affected by the subcarrier allocation across multiple Tx antennas. Thus, we propose a new subcarrier allocation strategy that efficiently maximizes the achievable rate and simultaneously reduces the MSE of the radar images.

In the proposed BMP-based radar imaging, the received signal at the MIMO OFDM RadCom platform is first transformed into frequency domain to take symbol-based signal processing (i.e., spectral division operation [15]) and then reformulated in terms of the (azimuth angle, range) patches in the image region of interest. We can then formulate the radar image estimation problem, where the maximum a posterior (MAP) estimate is obtained. In addition, we consider that the RadCom platform transmits information data through OFDM waveforms to the information receiver with multiple antennas, where MIMO channel is established from RadCom platform to the information receiver. Therefore, the achievable rate at the information receiver can be regarded as the communication performance for the RadCom platform. To understand the impact of the subcarrier allocation on the MSE, we analyze the lower bound of Cramer–Rao lower bound (CRLB) in our radar image estimation with three different subcarrier allocation methods—equi-space subcarrier allocation, block-wise subcarrier allocation, and pseudorandom subcarrier allocation. Based on the analysis, we propose a new subcarrier allocation strategy that efficiently maximizes the achievable rate and simultaneously reduces the MSE of the radar images. We also discuss how the Doppler frequency of the targets in 2D radar image can be estimated by exploiting the output of our radar image estimation problem. Through computer simulations, our BMP-based method exhibits radar images with higher resolution, compared to the conventional back-projection-based imaging method [16]. In addition, it is also verified that through the proposed subcarrier allocation strategy, a high achievable rate is obtained without sacrificing the radar imaging performance.

The rest of this paper is organized as follows. In Section 2, the system model for MIMO OFDM RadCom platform is introduced. In Section 3, the BMP-based radar imaging with MIMO OFDM waveform is proposed, where the received signal is processed with the frequency-domain OFDM signal processing at RadCom platform. Then, it is reformulated in terms of the (azimuth, range) patches and is cast in the sparse radar image reconstruction problem. In Section 4, information transfer using MIMO OFDM waveform is explained and the achievable rate maximizing transmission strategy is developed. In Section 5, the subcarrier allocation strategy considering both the radar performance and the communication performance is proposed. In Section 6, the Doppler frequency estimation from the estimated 2D radar image results is presented. In Section 7 and Section 8, the simulation results and conclusion are presented.

## 2. System Model for Joint Radar and Communication with MIMO OFDM Waveform

**Figure 1:**
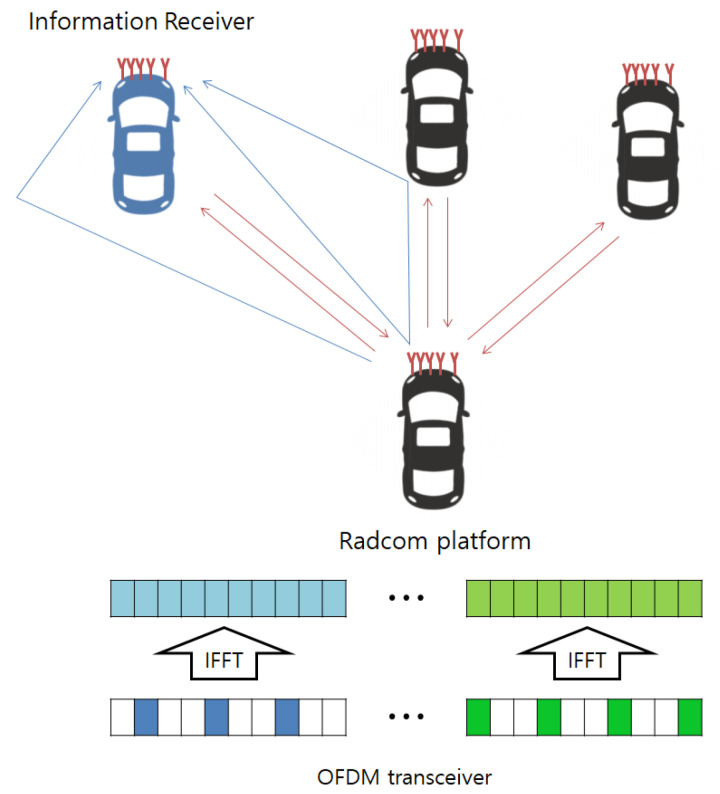
Multiple-input multiple-output (MIMO) orthogonal frequency division multiplexing (OFDM) RadCom system composed of co-located $M_{t}$ Tx antennas and $M_{r}$ radar Rx antennas.

We consider the MIMO OFDM RadCom system composed of co-located $M_{t}$ Tx antennas and $M_{r}$ radar Rx antennas as in Figure 1. Here, the OFDM waveform conveying the message information is transmitted through multiple Tx antennas to an information receiver with multiple antennas. In addition, the backscattered OFDM waveform received by multiple antennas at RadCom is exploited to generate the radar image in front.

### 2.1. Tx Signal Model at RadCom Tx Antenna

The OFDM signal transmitted through the $m_{t}$th antenna is generally given as [12,13,17]

(1) $$\;x_{m_{t}}\left(t\right)=\frac{1}{\sqrt{N_{c}}}\;\sum_{\mu =0}^{N_{sym}-1}\sum_{n_{c}\in {\bar{N}}_{m_{t}}}s\left[n_{c},\mu \right]e^{j2\pi f_{n_{c}}t}rect\left(\;\frac{t_{f}\;+\;T_{CP}}{T_{OFDM}}\;\right)\;,\;$$

where $N_{c}$ is the number of subcarriers in one OFDM symbol (equivalently, OFDM pulse) and $N_{sym}$ is the number of total transmitted OFDM symbols during the coherent processing interval. In addition, $s[n_{c},\mu ]$ is the quadrature amplitude modulated symbols carrying the information data with $E[∥s\left[n_{c},\mu \right]∥]=\frac{P_{tx}}{N_{c}}$; ${\bar{N}}_{m_{t}}$ is the set of subcarrier indices allocated to the $m_{t}$th Tx antenna; and the subcarrier frequency, $f_{n_{c}}$, is given as $f_{n_{c}}=n_{c}\Delta f$ with a intercarrier spacing Δf. In addition, $T_{CP}$ (resp., $T_{OFDM}$) is the time interval for one cyclic prefix (resp., one OFDM symbol) duration. Then, note that $T_{OFDM}=T+T_{CP}$ with T=1/Δf. In addition, by introducing the idle time $T_{IDLE}$ between the OFDM pulses, the symbol repetition interval can be set as $T_{SRI}=T_{OFDM}+T_{IDLE}$ Then, the continuous time *t* can be expressed as $t=t_{f}+\left(\mu -1\right)T_{SRI}$.

### 2.2. Received Signal Model at RadCom Rx Antenna

If the μth OFDM pulse backscattered from *K* targets are sampled with a sampling time, $T_{s}\left(=1\left(N_{c}\Delta f\right)\right)$ at the $m_{r}$th RadCom Rx antenna, the discrete-time received signal can then be given as

(2) $$\begin{array}{ccc} \;y[n_{d},m_{r},\mu ] & = & \sum_{k=1}^{K}\frac{\gamma_{k}}{\sqrt{N_{c}}}\sum_{m_{t}=1}^{M_{t}}\sum_{n_{c}\in {\bar{N}}_{m_{t}}}\;s\left[n_{c},\mu \right]e^{\frac{j2\pi f_{n_{c}}n_{d}}{N_{c}\Delta f}}e^{-j2\pi f_{n_{c}}\tau_{m_{r}m_{t}k}}e^{j2\pi f_{D}\mu T_{SRI}}+n_{r}\left[n_{d},m_{r},\mu \right], \end{array}$$

where $\tau_{m_{r}m_{t}k}$ is the delay time, for which the signal from the $m_{t}$th Tx antenna is reflected on the *k*th target ahead and received by the $m_{r}$th Rx antenna, and $f_{D}$ is the Doppler shift, given as $f_{D}=-\frac{2v_{k}f_{c}}{c}$, where $f_{c}$ is the carrier frequency and *c* is the speed of light. Here, $n_{r}\left[n_{d},m_{r},\mu \right]$ denotes the complex additive white Gaussian noise with a variance $\sigma_{n}^{2}$ (i.e., $n_{r}\left[n_{d},m_{r},\mu \right]∼\mathrm{CN}\;\left(0,\sigma_{n}^{2}\right)$). Assuming Tx/Rx antennas are co-located in a virtual linear array at RadCom [18], for the far-field target distance, $\tau_{m_{r}m_{t}k}$ can be given as

(3) $$\begin{array}{c} \tau_{m_{r}m_{t}k}=\frac{2}{c}R_{k0}+\frac{1}{c}d_{m_{t}m_{r}}\sin\theta_{k}, \end{array}$$

where $R_{k0}$ is the distance from the reference antenna element (i.e., the first element in the virtual antenna array at RadCom) to the *k*th target and $\theta_{k}$ is the azimuth angle of the *k*th target with respect to RadCom. By substituting (3) into (2), it can be given as

(4) $$\begin{array}{ccc} \;y[n_{d},m_{r},\mu ]\; & \;=\; & \;\sum_{k=1}^{K}\frac{\gamma_{k}}{\sqrt{N_{c}}}\sum_{m_{t}=1}^{M_{t}}\;\sum_{n_{c}\in {\bar{N}}_{m_{t}}}\;s\left[n_{c},\mu \right]e^{j2\pi f_{n_{c}}\frac{n_{d}}{N_{c}}T}e^{-j2\pi f_{n_{c}}\tau_{0k}}e^{-j2\pi \frac{1}{\lambda }d_{m_{t}m_{t}}\sin\theta_{k}}e^{j2\pi f_{D_{k}}\mu T_{SRI}}\\ & & +n_{r}\left[n_{d},m_{r},\mu \right], \end{array}$$

where $\tau_{0k}=\frac{2R_{k0}}{c}$. In (2) and (4), $\gamma_{k}$ is the coefficient aggregating the target reflection gain and the path-loss. Specifically, with the antenna gain, *G*, and the reflection gain of the *k*th target, ${\bar{\gamma }}_{k}$, $\gamma_{k}$ is given as

(5) $$\begin{array}{c} \gamma_{k}=\frac{G}{{\left(R_{km_{t}}\right)}^{2}{\left(R_{km_{r}}\right)}^{2}}{\bar{\gamma }}_{k}\approx \frac{G}{{\left(R_{k0}\right)}^{4}}{\bar{\gamma }}_{k}, \end{array}$$

where ${\bar{\gamma }}_{k}$ follows a complex Gaussian distribution with a zero-mean and a unit variance (that is, ${\bar{\gamma }}_{k}∼\mathrm{CN}\left(0,1\right)$). In (5), the last approximation is from the assumption of the far-field target distance.

Note that to form a virtual linear array with $M_{t}M_{r}$ elements at RadCom, the OFDM signals transmitted from different Tx antennas should be orthogonal at each RadCom receiver, which implies that the subcarriers should be exclusively allocated to different Tx antennas, that is,

(6) $$\begin{array}{c} {\bar{N}}_{m_{t}}\cap {\bar{N}}_{m_{t}^{\prime }}=\phi ,\;\mathrm{for}m_{t}\neq m_{t}^{\prime }. \end{array}$$

### 2.3. Received Signal Model at Information Rx Antenna

When the information receiver with $M_{r}$ Rx antennas receives $N_{p}$ independent multipaths of the OFDM signal (1), the received signal can be given as

(7) $$\begin{array}{c} \;y_{m_{r},I}\left(t\right)\;=\;\sum_{n_{p}=1}^{N_{p}}\;\sum_{m_{t}=1}^{M_{t}}\;h_{m_{r},m_{t},n_{p}}x_{m_{t}}\left(t-\tau_{m_{r},m_{t},n_{p}}\right)+n_{I}\left(t\right),\; \end{array}$$

where $h_{m_{r},m_{t},n_{p}}$ is the path gain of the $n_{p}$th multipath between the $m_{t}$th Tx antenna and the $m_{r}$th information Rx antenna, and $\tau_{m_{r},m_{t},n_{p}}$ is the associated multipath delay. After sampling and CP removal, the discrete-time received signal can be given as

(8) $$\begin{array}{c} \;\;{\tilde{y}}_{m_{r},I}\left[n_{d}\right]\;=\;\sum_{m_{t}=1}^{M_{t}}\;\sum_{n_{c}\in {\bar{N}}_{m_{t}}}\;\;H_{m_{r},m_{t}}\;\left[n_{c}\right]s\left[n_{c}\right]e^{j2\pi f_{n_{c}}\frac{n_{d}}{N_{c}}T}\;+\;{\tilde{n}}_{I}\left[n_{d}\right]\;, \end{array}$$

where $H_{m_{r},m_{t}}\left[n_{c}\right]$ is the frequency selective channel gain associated with the $n_{c}$th subcarrier between the $m_{t}$th Tx antenna and the $m_{r}$th information Rx antenna, which can be obtained from the DFT of $h_{m_{r},m_{t}}\left[n\right]=\sum_{n_{p}=1}^{N_{p}}h_{m_{r},m_{t},n_{p}}\delta \left[n-n_{m_{r},m_{t},n_{p}}\right]$ with $n_{m_{r},m_{t},n_{p}}=\left\lceil \tau_{m_{r},m_{t},n_{p}}/T_{s}\right\rfloor$. Note that the symbol index μ is omitted for the notation simplicity.

## 3. Compressive Sensing Based MIMO OFDM Radar Imaging Algorithm

### 3.1. Signal Reformulation

To apply the compressive sensing algorithm, the received signal in (4) is expressed in vector form as

(9) $$\begin{array}{c} y_{t,m_{r}}^{\mu }=\left[\begin{array}{c} y[0,m_{r},\mu ]\\ y[1,m_{r},\mu ]\\ \vdots \\ y[N_{c}-1,m_{r},\mu ]] \end{array}\right]. \end{array}$$

By taking DFT on $y_{t,m_{r}}^{\mu }$, we can have (10), as shown at the top of the next page. Here, the Doppler shift causes the intercarrier interference and the associated performance degradation in both radar and communication performances. Specifically, the intercarrier interference degrades the coefficient aggregating the target reflection gain for the radar imaging and the effective channel gain for the communication. To overcome the performance degradation, the subcarrier spacing (Δf) can be chosen much larger than the maximum Doppler shift in [17]. In [19], the authors have proposed the all-cell Doppler correction algorithm in which the intercarrier interference is corrected by transmitting the repeated OFDM symbols. Motivated by the work in [19], the compensation of the intercarrier interference due to the Doppler shift can be developed for our RadCom system.

(10) $$\begin{array}{c} y_{f,m_{r}}^{\mu }=F_{N_{c}}y_{t,m_{r}}^{\mu }=\left[\begin{array}{c} \sum_{k=1}^{K}\frac{\gamma_{k}}{\sqrt{N_{c}}}s\left[0,\mu \right]\sum_{m_{t}=1}^{M_{t}}\Omega_{0,m_{t}}e^{-j2\pi \frac{d_{m_{t}m_{t}}}{\lambda }\sin\theta_{k}}e^{j2\pi f_{D_{k}}\mu T_{SRI}}\\ \sum_{k=1}^{K}\frac{\gamma_{k}}{\sqrt{N_{c}}}s\left[1,\mu \right]\sum_{m_{t}=1}^{M_{t}}\Omega_{1,m_{t}}e^{-j2\pi \Delta f\tau_{k0}}e^{-j2\pi \frac{d_{m_{t}m_{t}}}{\lambda }\sin\theta_{k}}e^{j2\pi f_{D_{k}}\mu T_{SRI}}\\ \vdots \\ \sum_{k=1}^{K}\frac{\gamma_{k}}{\sqrt{N_{c}}}s\left[N_{c}-1,\mu \right]\sum_{m_{t}=1}^{M_{t}}\Omega_{N_{c}-1,m_{t}}e^{-j2\pi \left(N_{c}-1\right)\Delta f\tau_{k0}}e^{-j2\pi \frac{d_{m_{t}m_{t}}}{\lambda }\sin\theta_{k}}e^{j2\pi f_{D_{k}}\mu T_{SRI}} \end{array}\right]+n_{r}, \end{array}$$

where $F_{N_{c}}$ is a DFT matrix in which the (i,j)th element is given as $\frac{1}{\sqrt{N_{c}}}e^{-j2\pi \left(i-1\right)\left(j-1\right)/N_{c}}$, and $\Omega_{n_{c},m_{t}}$ denotes an indicator function such that $\Omega_{n_{c},m_{t}}=1$ for $n_{c}\in {\bar{N}}_{m_{t}}$, and $\Omega_{n_{c},m_{t}}=0$ otherwise.

**Figure 2:**
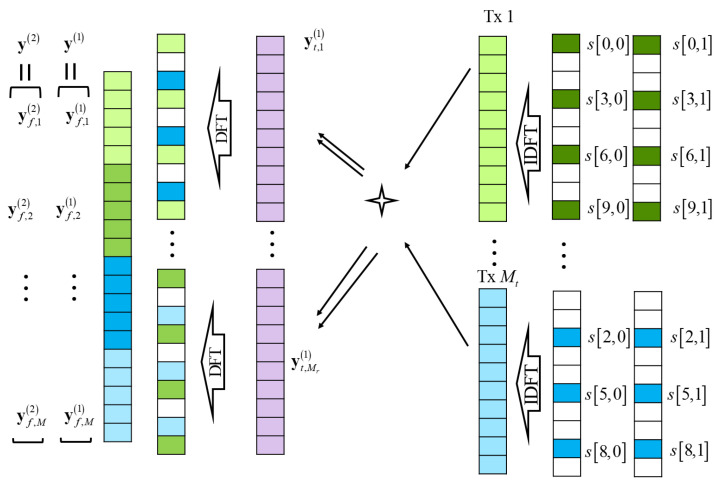
Radar signal process in MIMO OFDM RadCom.

Thanks to (6), the signals transmitted from different transmit antennas are separated at each receiver. Accordingly, $y_{f,m_{r}}^{\mu }$ in (10) can be stacked as

(11) $$\begin{array}{c} y^{\mu }=\left[\begin{array}{c} y_{f,1}^{\mu }\\ y_{f,2}^{\mu }\\ \vdots \\ y_{f,M}^{\mu } \end{array}\right], \end{array}$$

where the virtual antenna element index *m* is used as $m=1,\ldots ,M(=M_{t}M_{r})$ instead of the antenna indices $\left(m_{t},m_{r}\right)$. The overall process is briefly described in Figure 2.

**Figure 3:**
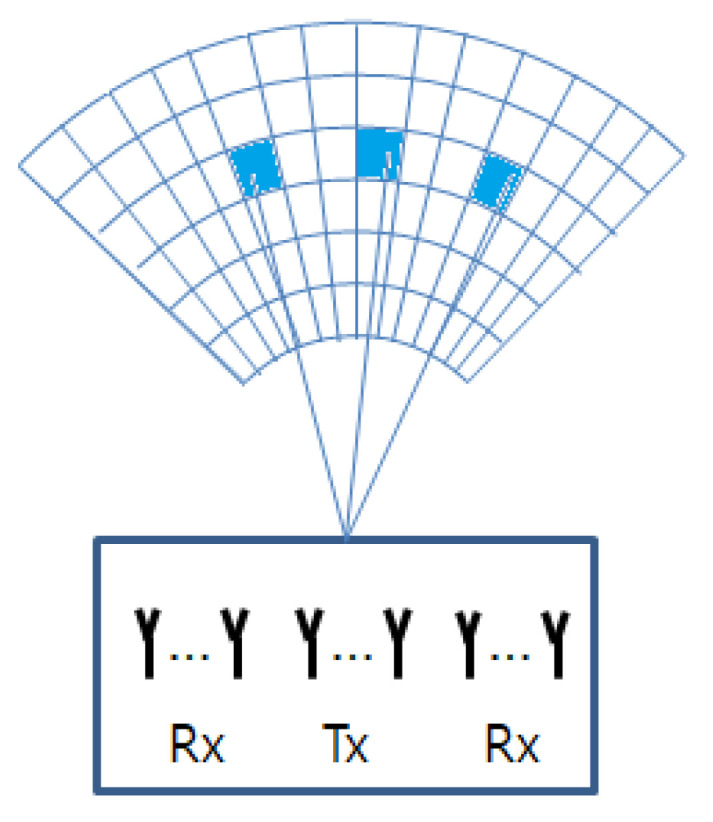
The R×P two-dimensional patches (range × azimuth angle).

From (10), the elements in (11) can be given as the linear sum of the aggregated reflection coefficients (i.e., $\gamma_{k}$) backscattered from multiple targets. Accordingly, by dividing the image region of interest into R×P two-dimensional patches (range × azimuth angle) as in Figure 3, the $n_{c}+N_{c}\left(m-1\right)$th element of $y^{\mu }$ can be expressed as

(12) $$\begin{array}{c} \;{\left[y^{\mu }\right]}_{n_{c}+N_{c}\left(m\;-\;1\right)}=\sum_{p=1}^{P}\sum_{r=1}^{R}x\left(r,p\right)\frac{1}{\sqrt{N_{c}}}s\left[n_{c},\mu \right]e^{-j2\pi n_{c}\tau \left(m,r,p\right)}e^{j2\pi f_{D(r,p)}\mu T_{SRI}}+n_{r}\left[n_{c},m,\mu \right],\; \end{array}$$

where $x\left(r,p\right)$ is a coefficient aggregating the target reflection gain, the antenna gain, and the path-loss when the associated target is on (r,p)th patch. That is, $x\left(r,p\right)=\gamma_{k}$ if the *k*th target is in the (r,p)th patch; otherwise, x(r,p)=0. In addition,

$$\tau \left(m,r,p\right)=\frac{2}{c}R_{r}+\frac{1}{c}\left(d\left(m-1\right)\sin\theta_{p}\right),m=1,\ldots ,M,$$

where $\left(R_{r},\theta_{p}\right)$ are the range and the azimuth angle of the (r,p)th patch with respect to the RadCom.

In what follows, understanding that the radar image can be obtained from a single backscattered OFDM pulse, we set the pulse index μ as 0 and omit the pulse index μ (To estimate the velocities of targets, multiple OFDM pulses should be coherently processed, which is discussed in Section 6). Then, $y^{\mu }$ in (11) can be rewritten as

(13) $$\begin{array}{c} y=Ax+n, \end{array}$$

where A is given as

(14) $$\begin{array}{c} A=\left[\begin{array}{cccc} A(1,1,1,1) & A(1,1,1,2) & \ldots & A(1,1,R,P)\\ A(2,1,1,1) & A(2,1,1,2) & \ldots & A(2,1,R,P)\\ \vdots & \vdots & \ddots & \vdots \\ A(M,N,1,1) & A(M,N,1,2) & \ldots & A(M,N,R,P) \end{array}\right], \end{array}$$

and $x={\left[x(1,1),x(1,2),\ldots ,x(R,P)\right]}^{T}\in C^{PR\times 1}$. Here, *N* is given as $N=N_{c}/M_{t}$ with the assumption that each Tx antenna is assigned the same number of subcarriers. Then, A(m,n,r,p) is given as $A\left(m,n,r,p\right)=\frac{1}{N_{c}}s\left[n_{c}\left(m,n\right)\right]e^{-j2\pi n_{c}\left(m,n\right)\tau \left(m,r,p\right)}$, where $n_{c}\left(m,n\right)$ denotes the subcarrier index of the *n*th element in ${\bar{N}}_{m_{t}}$ associated with the *m*th virtual array antenna element.

In (14), the row size, MN, is associated with the number of measurement samples and the column size, PR, is associated with the number of patches in the radar image region of interest. However, PR≫MN≫1 for general high-resolution radar imaging problems. Fortunately, when x is sparse and the matrix A in (14) satisfies the restricted isometry property [20], the solution of (13) can be obtained by applying the compressive sensing approach and the associated compressive sensing problem is formulated as

(15) $$\begin{array}{c} \hat{x}=\underset{x}{\arg\min}{\left\|x\right\|}_{0}-{\left\|y-\mathrm{Ax}\right\|}_{2}^{2}\leq \epsilon . \end{array}$$

### 3.2. BMP-Based MIMO OFDM Radar Imaging

Thanks to the reasonable computational complexity with high immunity to noise, the BMP algorithm has been widely applied to the compressive sensing problems [21]. Especially, BMP-based radar imaging has been proposed for the distributed FMCW MIMO radar system. Motivated by the work in [14], BMP-based MIMO OFDM radar imaging is developed by applying BMP to (13).

For the BMP algorithm, we first define a new PR×1 binary support vector:

$$z={\left[z(1,1),z(1,2),\ldots ,z(R,P)\right]}^{T}\in {\left\{0,1\right\}}^{PR},$$

where z(r,p)=1 only when a target is at the (r,p)th patch and otherwise, z(r,p)=0. Throughout the paper, it is assumed that P(z(r,p)=1)=c with a constant *c* for r=1,…,R and p=1,…,P.

From (5), x|z∼CN(0,R(z))), where R(z) is given as

(16) $$\begin{array}{c} R\left(z\right)=G\times diag\left\{\frac{z(1,1)}{R_{1}^{4}},\frac{z(1,2)}{R_{1}^{4}},\ldots ,\frac{z(R,P)}{R_{R}^{4}}\right\}, \end{array}$$

where $diag\left\{a_{1}\ldots ,a_{N}\right\}$ denotes a diagonal matrix having its diagonal elements as $a_{1},\ldots ,a_{N}$. In the BMP algorithm [14], x can be iteratively updated such as

(17) $$\begin{array}{c} \hat{x}=\underset{x}{\arg\max}\;p\left(x|y\right)=\underset{x}{\arg\max}\sum_{z}p\left(x|y,z\right)p\left(z|y\right). \end{array}$$

Specifically, from the works in [14,22], the BMP for the radar imaging can be mainly decomposed as two steps: (1) update of z and (2) update of x for a given z. Here, in the update of z, it is updated by maximizing the posterior probabilities, p(z|y). In the update of x for a given z, it is updated such that p(x|y,z) is maximized.

We note that in the update of z, by defining ψ(z)≜lnp(y|z), the non-zero element of z can be found such that the updated z maximizes ψ(z). This is because p(z|y)∝p(y|z)p(z), and p(z) is a constant for all patches. Here, from (13) and (16), it can be found that y|z also follows the complex Gaussian distribution with a zero-mean and covariance matrix Φ(z), which is given as

(18) $$\begin{array}{c} \Phi \left(z\right)=AR\left(z\right)A^{H}+\sigma_{n}^{2}I_{MN}. \end{array}$$

Accordingly, ψ(z) can be then derived as

(19) $$\begin{array}{c} \psi \left(z\right)=-MN\ln\pi -\ln\det\left(\Phi \left(z\right)\right)-y^{H}\Phi^{-1}\left(z\right)y. \end{array}$$

In [22], to find the nonzero elements of z that maximize Φ(z), a tree search-based support update algorithm has been proposed, and it can also be applied to our problem. For compactness, a brief sketch of the algorithm is provided in what follows.

We first initialize $z^{\left(0\right)}$ as $0_{PR}$, and then $\psi (z_{k}^{\left(1\right)})$ is evaluated from (19) for k=1,…,PR, where $z_{k}^{\left(1\right)}$ is obtained by setting 1 at the *k*th element of $z^{\left(0\right)}$. Then, by keeping $z_{k}^{\left(1\right)}$ associated with the *D* largest $\psi (z_{k}^{\left(1\right)})$ as the possible candidate set ${\bar{Z}}^{\left(1\right)}$, the next nonzero element of support vector can be found by evaluating $\psi (z_{k}^{\left(2\right)})$, where $z_{k}^{\left(2\right)}$ is obtained by changing one element of $z_{k}^{\left(1\right)}\in Z^{\left(1\right)}$ into 1. Here, $z_{k}^{\left(2\right)}$ associated with the *D* largest $\psi (z_{k}^{\left(2\right)})$ are collected at the set of ${\bar{Z}}^{\left(2\right)}$. This process is repeated until the stopping criterion is satisfied. Here, the stopping criterion can be set to terminate when the estimation error $\epsilon^{\left(i\right)}≜{∥y^{\psi u}-A{\hat{x}}^{\left(i\right)}∥}_{|z=z_{\bar{k}}}^{2}$ is less than a preset threshold value, $\epsilon_{th}$. Here, $z_{\bar{k}}$ is given as $z_{\bar{k}}=\underset{z_{k}}{\arg\max}\;\psi \left(z_{k}^{\left(i\right)}\right)$ and ${\hat{x}}^{\left(i\right)}$ is an estimate of (13) for a given $z_{\bar{k}}$.

In the coefficient update, the coefficient x is updated such that the posterior probability p(x|y) in (17) is maximized when $\tilde{z}$ is given. By taking a similar approach as in [14], it can be derived that the estimate $\hat{x}$ that maximizes (13) is given as

(20) $$\begin{array}{c} \hat{x}={\left(\sigma_{n}^{2}R^{-1}\left(\tilde{z}\right)+A_{\tilde{z}}^{H}A_{\tilde{z}}\right)}^{-1}A_{\tilde{z}}^{H}y, \end{array}$$

where $A_{\tilde{z}}=Adiag\left\{\tilde{z}\right\}$. After several iterations of the process described above, the solution for (13) can be obtained as (20). For the details, refer to the work in [14].

## 4. Information Transfer Using MIMO OFDM Waveform

For information transfer, the QAM signal is carried on the subcarriers of the transmitted OFDM waveform as in (1), and to demodulate QAM signal, the discrete-time received signal for the $m_{r}$th information Rx antenna in (8) should be transformed into the frequency domain as

(21) $$\begin{array}{ccc} {\tilde{y}}_{m_{r},I}\; & \;=\; & \;F_{N_{c}}\left[\begin{array}{c} {\tilde{y}}_{m_{r},I}\left[0\right]\\ {\tilde{y}}_{m_{r},I}\left[1\right]\\ \vdots \\ {\tilde{y}}_{m_{r},I}\left[N_{c}-1\right] \end{array}\right]\\ & \;=\;\; & \;\;\left[\;\begin{array}{c} s\left[0\right]\sum_{m_{t}}^{M_{t}}\Omega_{0,m_{t}}H_{m_{r},m_{t}}\left[0\right]\\ s\left[1\right]\sum_{m_{t}}^{M_{t}}\Omega_{1,m_{t}}H_{m_{r},m_{t}}\left[1\right]\\ \vdots \\ s\left[N_{c}\;-\;1\right]\sum_{m_{t}}^{M_{t}}\Omega_{N_{c}\;-\;1,m_{t}}H_{m_{r},m_{t}}\left[N_{c}\;-\;1\right]\; \end{array}\;\right]\;+{\tilde{n}}_{I}. \end{array}$$

To maximize the achievable rate at the information receiver, the maximal ratio combining (MRC) scheme is used at each subcarrier. Specifically, by denoting $H_{m_{t}}\left[n_{c}\right]={\left[H_{1,m_{t}}\left[n_{c}\right],H_{2,m_{t}}\left[n_{c}\right]\ldots ,H_{M_{r},m_{t}}\left[n_{c}\right]\right]}^{T}$, the MRC output can be given as the weighted sum of the frequency-domain received signals with a weight vector, $w_{m_{t}}\left[n_{c}\right]=\frac{H_{m_{t}}\left[n_{c}\right]}{∥H_{m_{t}}\left[n_{c}\right]∥}$. The output of the MRC can then be given as

(22) $$\begin{array}{ccc} y_{I}\; & \;=\; & \;\left[\;\begin{array}{c} s\left[0\right]\sum_{m_{t}}^{M_{t}}\Omega_{0,m_{t}}∥H_{m_{t}}\left[0\right]∥\\ s\left[1\right]\sum_{m_{t}}^{M_{t}}\Omega_{1,m_{t}}∥H_{m_{t}}\left[1\right]∥\\ \vdots \\ s\left[N_{c}\;-\;1\right]\sum_{m_{t}}^{M_{t}}\Omega_{N_{c}\;-\;1,m_{t}}∥H_{m_{t}}\left[N_{c}\;-\;1\right]∥\; \end{array}\;\right]\;+n_{I}. \end{array}$$

From (6) and (21), the achievable rate at the information receiver can be derived as

(23) $$\begin{array}{ccc} R_{I} & = & \sum_{i=0}^{N_{c}-1}\log\left(1+\frac{(\sum_{m_{t}=1}^{M_{t}}\Omega_{i,m_{t}}∥H_{m_{t}}\left[i\right]{∥)}^{2}P_{tx}}{\sigma_{n}^{2}}\right)\\ & = & \sum_{i=0}^{N_{c}-1}\sum_{m_{t}=1}^{M_{t}}\log\left(1+\frac{\Omega_{i,m_{t}}{∥H_{m_{t}}\left[i\right]∥}^{2}P_{tx}}{\sigma_{n}^{2}}\right). \end{array}$$

We note that the pilot frequency selection does not affect the radar imaging, because the transmit symbols are known at the RadCom platform. In contrast, the pilot frequency selection affects the channel estimation at the information receiver [23]. Even though we analyze the theoretic achievable rate, assuming the perfect channel state information at the receiver, by focusing on the effect of the subcarrier allocation on both radar and communication performances in Section 5, the analysis of the achievable rate considering the pilot frequency selection and the channel estimation error is an important topic but is out of scope of this paper.

## 5. Subcarrier Allocation Strategy

We note that both the radar imaging and the communication performances are dependent on how the subcarriers are allocated to multiple Tx antennas. In our RadCom system, the MSE of radar image in (13) (i.e., $E[∥\hat{x}-x{∥}^{2}]$) and the achievable rate in (23) are, respectively, considered as the radar imaging and the communication performance measures, and the subcarrier allocation method is equivalent with the determination of $\Omega_{i,m_{t}}$ for $i=0,\ldots ,N_{c}-1$ and $m_{t}=1,\ldots M_{t}$ under the constraint (6).

For the communication performance, the achievable rate in (23) can be maximized by allocating each subcarrier to the Tx antenna with the maximum channel gain on that subcarrier. That is, the achievable rate maximization strategy can be given as

(24) $$\begin{array}{c} \Omega_{i,{\bar{m}}_{t}}=1\;\mathrm{for}\;{\bar{m}}_{t}=\underset{m_{t}=1,\ldots ,M_{t}}{\arg}\max||H_{m_{t}}\left[i\right]\left|\right|, \end{array}$$

and $\Omega_{i,m_{t}}=0$ otherwise. However, when the coherent channel bandwidth is large (i.e., the delay spread is small), subcarriers may be intensively allocated only to specific Tx antennas, which is not desirable to the radar imaging. In extreme case, if all subcarriers are allocated only to a single Tx antenna, the azimuth angle resolution disappears.

Note that the matrix A in (13) is implicitly dependent on the subcarrier allocation, but its effect on the MSE of radar image is subtle and difficult to analyze. Instead, the CRLB of MSE is considered. In [24], assuming that the support set is known, the MSE of the compressive sensing problem is lower bounded by

(25) $$\begin{array}{c} CRLB_{z}=\sigma_{n}^{2}Trace\left({\left(A_{z}^{H}A_{z}\right)}^{-1}\right), \end{array}$$

where $A_{z}$ is a sub-matrix of A that consists of the columns associated with the indices of z. Unfortunately, it is still computationally prohibited to find optimal $\Omega_{i,m_{t}}$ for $i=0,\ldots ,N_{c}-1$ and $m_{t}=1,\ldots M_{t}$ such that (25) is minimized.

To shed light on the idea, in Table 1, the average CRLBs for the three different subcarrier allocation methods (equi-space, block-wise, and pseudorandom methods) with random support sets z are listed, when $N_{c}=256$, $M_{t}=M_{r}=4$, P=R=41, Δf=100kHz, and $\sigma_{n}^{2}=1$. In the equi-space subcarrier allocation, the $\left(m_{t}+M_{t}\times \left(j-1\right)-1\right)$th subcarriers, $j=1,\ldots ,N_{c}/M_{t}$, are allocated to the $m_{t}$th Tx antenna. In the block-wise subcarrier allocation, the $\left(j-1+N_{c}/M_{t}\times \left(m_{t}-1\right)\right)$th subcarriers, $j=1,\ldots ,N_{c}/M_{t}$, are allocated to the $m_{t}$th Tx antenna. Note that, for simplicity, it is assumed that $N_{c}$ is a multiple of $M_{t}$. In the pseudorandom subcarrier allocation, the $N_{c}/M_{t}$ subcarriers are randomly allocated to each Tx antenna. From Table 1, the equi-space allocation method exhibits lower CRLB than other methods, which implies that when the subcarriers are regularly allocated to Tx antennas, A in (13) can project the sparse x onto the received signal $y^{\mu }$ and reduce the MSE; that is, it is advantageous to keep the frequency spacing some distance apart for the subcarriers allocated to the same Tx antennas.

Motivated from the above observations, the subcarrier allocation strategy considering both the communication and radar imaging performances is proposed. Here, to avoid unbiased allocation of subcarriers to a specific antenna, the number of subcarriers allocated to each antenna is set to have the same number. That is, $N_{count,m_{t}}=\frac{N_{c}}{M_{t}}$ for $m_{t}=1,\ldots ,M_{t}$. To guarantee the frequency spacing for the radar imaging, $N_{c}$ subcarriers are first divided into multiple sub-blocks with $\alpha N_{c}$ subcarriers, where α∈(0,1] such that $\alpha N_{c}$ and $\frac{\alpha N_{c}}{M_{t}}$ are integers (For ease of explanation, we confine the value of α, but it can be extended to the general cases that $\alpha N_{c}$ and $\frac{\alpha N_{c}}{M_{t}}$ are not integers). Then, the number of the sub-block is given as $N_{blk}=1/\alpha$, and the subcarriers within the same sub-block are allocated to the Tx antenna so that its associated channel gain is maximized, which is done similarly in (24). By defining $H\in R^{M_{t}\times N_{c}}$ as

(26) $$\begin{array}{c} H=\left[\begin{array}{cccc} \left|\right|H_{1}\left[0\right]\left|\right| & \left|\right|H_{1}\left[1\right]\left|\right| & \cdots & \left|\right|H_{1}\left[N_{c}-1\right]\left|\right|\\ \left|\right|H_{2}\left[0\right]\left|\right| & \left|\right|H_{2}\left[1\right]\left|\right| & \cdots & \left|\right|H_{2}\left[N_{c}-1\right]\left|\right|\\ \vdots & \ddots & & \vdots \\ \left|\right|H_{M_{t}}\left[0\right]\left|\right| & \left|\right|H_{M_{t}}\left[1\right]\left|\right| & \cdots & \left|\right|H_{M_{t}}\left[N_{c}-1\right]\left|\right| \end{array}\right], \end{array}$$

the above explained process is summarized as Algorithm 1.

**Figure 4:**
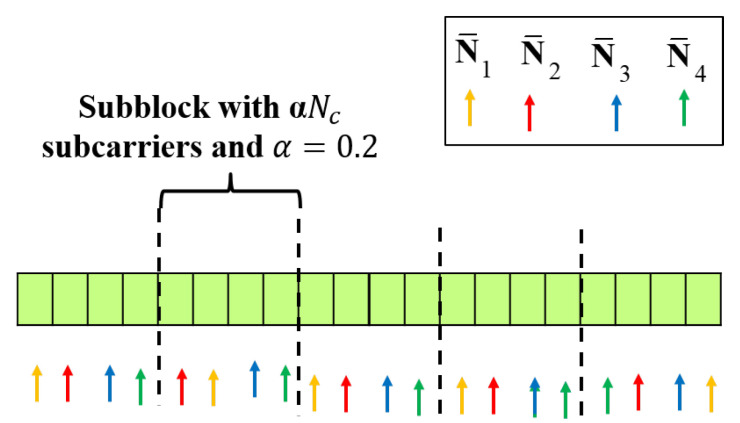
An example of the operation of the proposed subcarrier allocation strategy with $N_{c}=20$, α=0.2, and $M_{t}=4$.

In Figure 4, an example of the operation of the proposed strategy is shown when $N_{c}=20$, α=0.2, and $M_{t}=4$. Within the same block, the subcarriers are allocated such that the achievable rate is maximized. Furthermore, it is possible to ensure a frequency space of at least $\alpha N_{c}$ on average between subcarriers from different sub-blocks allocated to the same Tx antenna. Specifically, by dividing the subcarriers into multiple sub-blocks, the subcarriers within the same sub-block can be allocated to the Tx antenna so that its associated channel gain is maximized. In addition, limiting the number of subcarriers that can be allocated to the same antenna within the same sub-block prevents consecutive subcarriers from being assigned to a specific antenna. We note that the proposed radar imaging and the subcarrier allocation methods can be applied to the OFDM waveforms having the null bands without difficulty.

**Algorithm 1** Subcarrier allocation method for a given α
1:
$N_{blk}\leftarrow \frac{1}{\alpha }$
2:$N_{count,m_{t}}\leftarrow 0$ for $m_{t}=1,\ldots M_{t}$3:
$n_{blk}\leftarrow 0$
4:$\Omega_{i,m_{t}}\leftarrow 0$ for $i=0,\ldots ,N_{c}-1$, $m_{t}=1,\ldots M_{t}$5:
**do**
6:    $n_{blk}\leftarrow n_{blk}+1$7:    $H^{blk}=H\left[1:M_{t},\left(1:\alpha N_{c}\right)+\alpha N_{c}\left(n_{blk}-1\right)\right]$8:    **do**9:         $\left\{{\hat{m}}_{t},\hat{i}\right\}\leftarrow \underset{m_{t},i}{\arg\max}\;H^{blk}\left[m_{t},i\right]$10:        **if** $N_{count,{\hat{m}}_{t}}<\frac{\alpha N_{c}}{M_{t}}$ **then**11:           ${\hat{n}}_{c}\leftarrow i+N_{c}\left(n_{blk}-1\right)$12:           ${\hat{n}}_{c}\in {\bar{N}}_{{\hat{m}}_{t}}$, $N_{count,{\hat{m}}_{t}}\leftarrow N_{count,{\hat{m}}_{t}}+1$13:           $\Omega_{{\hat{n}}_{c},{\hat{m}}_{t}}\leftarrow 1$14:         **end if**15:        $H^{blk}\left[{\hat{m}}_{t},\hat{i}\right]\leftarrow 0$16:    **while** $N_{count,m_{t}}<\frac{\alpha N_{c}}{M_{t}}$ for any $m_{t}$17:
**while** $n_{blk}<N_{blk}$



Interestingly, for a large α, the subcarriers are more likely to be assigned to the Tx antenna with larger channel gains, but $\frac{\alpha N_{c}}{M_{t}}$ consecutive subcarriers within the same block may be allocated to the same Tx antenna in the worst case. Increasing α is advantageous in terms of communication performance, and vice versa. We also note that the case with α=1 is equivalent with the achievable rate maximization strategy in (24) with the constraint of the number of subcarriers per antenna (i.e., $N_{count,m_{t}}=\frac{N_{c}}{M_{t}}$ for $m_{t}=1,\ldots ,M_{t}$). Accordingly, the proposed subcarrier allocation method encompasses both communication and radar performances and, by adjusting α, the radar/communication performances can be properly balanced.

## 6. Discussion: Doppler Frequency Estimation

In Section 3, the 2D spatial radar image is obtained from a single backscattered MIMO OFDM pulse. To estimate the Doppler frequency of the target, multiple pulses should be collected in a coherent time duration. Accordingly, it is considered that $N_{sym}>1$. To estimate the Doppler frequencies of the targets detected in a 2D radar image, a FFT-based Doppler estimation method can be exploited.

By denoting the radar image estimate obtained from the μth radar echo signal (i.e., $y^{\mu }$) as ${\hat{x}}^{\left(\mu \right)}$, its nonzero element position corresponds to the target position in the 2D radar image from (12) and (13). Accordingly, the target location patch index set can be given as

(27) $$\begin{array}{c} \Phi =\left\{j|\sum_{\mu =0}^{N_{sym}-1}\left|{\left[{\hat{x}}^{\left(\mu \right)}\right]}_{j}\right|>\epsilon_{th}\right\}, \end{array}$$

where $\epsilon_{th}$ is a predefined threshold. Then, to estimate the Doppler frequency of the target associated with the patch *j* in Φ, the received signal is matched filtered, which is given as

$$y_{doppler,j}=\left[\begin{array}{c} {\left[A\right]}_{j}^{H}y^{\left(0\right)}\\ {\left[A\right]}_{j}^{H}y^{\left(1\right)}\\ \vdots \\ {\left[A\right]}_{j}^{H}y^{\left(N_{sym}-1\right)} \end{array}\right]=\alpha_{j}\left[\begin{array}{c} 1\\ e^{j2\pi f_{\nu_{j}}T_{SRI}}\\ \vdots \\ e^{j2\pi f_{\nu_{j}}\left(N_{sym}-1\right)T_{SRI}} \end{array}\right]+IN_{j},$$

where $\alpha_{j}={\left[x\right]}_{j}M_{r}P_{tx}$, and $IN_{j}$ is the residual term containing the received echo signal not co-phased with the *j*th column of A (i.e., ${\left[A\right]}_{j}$) and the noise. Then, the Doppler frequency of the patch *j* can be estimated by using FFT operation as

(28) $$\begin{array}{c} {\hat{f}}_{j}=\left[\underset{i}{\arg\max}\;F_{N_{sym}}{\bar{y}}_{doppler,j}\right]\times \frac{1}{N_{sym}T_{SRI}}Hz, \end{array}$$

and the associated velocity is given by $v_{j}=-\frac{c}{2}\frac{{\hat{f}}_{j}}{f_{c}}$.

## 7. Simulation Results

Computer simulations have been performed to verify the proposed scheme. In the simulations, the number of subcarriers is set as $N_{c}=256$ with the subcarrier space, Δf=100 kHz, and the carrier frequency ($f_{c}$) is given as 30 GHz. In addition, $T_{OFDM}$ = 12.5 s and $T_{SRI}$ = 50 s. In addition, the received SNR is defined as

(29) $$\begin{array}{c} SNR_{rec}=\frac{\sum_{k=1}^{K}{\left|\gamma_{k}\right|}^{2}}{K\sigma_{n}^{2}}. \end{array}$$

where $\sigma_{n}^{2}$ is the variance of the Gaussian noise.

**Figure 5:**
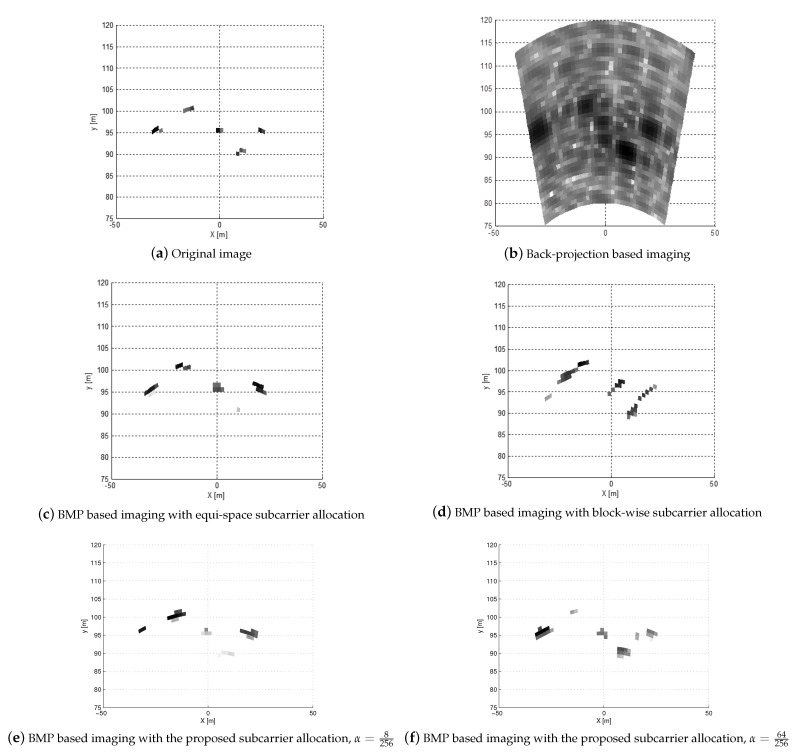
Radar images when MIMO OFDM radar is exploited with $M_{t}=4$ and $M_{r}=4$ at $SNR_{rec}$ = 14 dB.

In Figure 5, the radar images obtained from the proposed BMP-based MIMO OFDM radar imaging algorithm at $SNR_{rec}=14$ dB are displayed. For the reference, in Figure 5a, the original image is also provided. Specifically, five targets are in the radar image region of interest ($\left[80,120\right]\;m\times {\left[-20,20\right]}^{\circ }$), and each target consists of 85 point scatterers with a size of 4 m × 1 m. In addition, the numbers of the Tx and Rx antennas at the RadCom are set as $M_{t}=4$ and $M_{r}=4$, respectively. The size of each patch is given as $1\;m\times 0.5^{\circ }$, which implies that R=41 and P=41. If we decrease the pixel size, we can improve the image resolution, but the number of elements in x in (13) increases. Accordingly, the number of measurements (i.e., the dimension of y) should be increased proportional to the number of nonzero elements in x.

In Figure 5b, the radar image obtained by the back-projection method (i.e., ${\hat{x}}_{bp}=A^{H}y$) is shown. From the figure, the back-projection method exhibits poor resolution in both azimuth and range directions, that is, the targets in the radar image are severely blurred. The radar images of the BMP-based MIMO OFDM radar imaging with equi-space subcarrier allocation and block-wise subcarrier allocation are, respectively, shown in Figure 5c,d. It can be found that the BMP-based imaging with equi-space subcarrier allocation gives a better target image than back-projection method. However, when block-wise subcarrier allocation is exploited, some distorted target pixels can be found. In Figure 5e,f, the radar images of the BMP-based MIMO OFDM radar imaging with the proposed subcarrier allocation with $\alpha =\{\frac{8}{256},\frac{64}{256}\}$ are given. From the figures, when the proposed subcarrier allocation method is exploited, five targets are more clearly found, compared to that with the block-wise subcarrier allocation.

**Figure 6:**
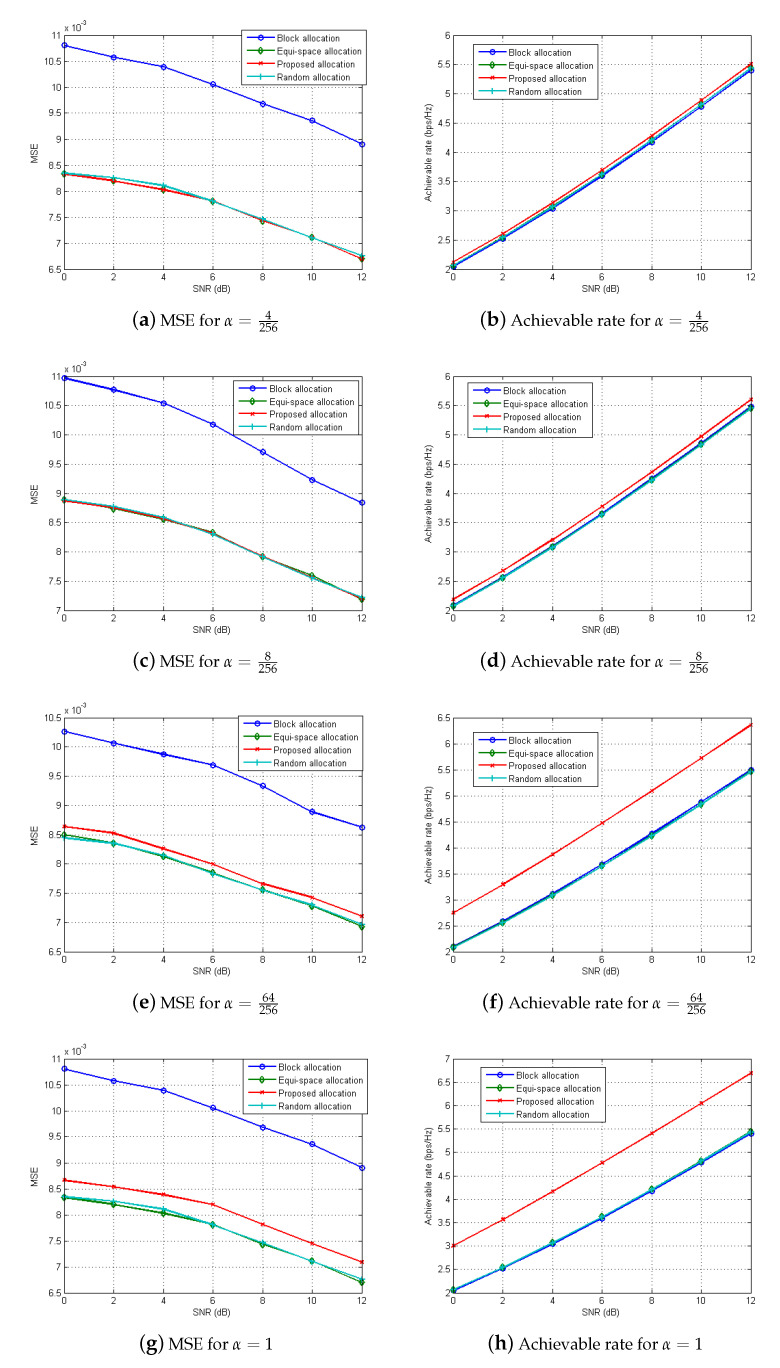
Mean square error (MSE) (**left**) and Achievable rate (**right**) for different subcarrier allocation methods.

To evaluate the performance of the proposed subcarrier allocation strategy for the RadCom, Monte Carlo simulations are also carried out with various α in Figure 6. Here, the number of multipaths is set as $N_{p}=8$ for the information channel link, and each path is set as a zero-mean complex Gaussian random variable with a unit variance. For the RadCom performance, we evaluate the MSE of the radar image, given as $E\left[∥\hat{x}{-x∥}^{2}\right]$, and the achievable rate at the information receiver. For comparison purpose, the performances of three different allocation methods (equi-space, block-wise, and pseudorandom methods) are also evaluated. From the figure, block-wise subcarrier allocation method shows the worst MSE performance, while equispace and pseudorandom subcarrier allocation methods have similar MSE performances, which coincides with the discussion in Section 5; that is, when subcarriers are evenly allocated across the entire band for each antenna, the radar performance can be improved. We note that the achievable rates of those three methods are similar, because they do not consider the communication performance at all.

From the figure, it can be found that when α increases, both the MSE and the achievable rate of radar images increase. That is, for small α, subcarriers for each antenna tend to be evenly allocated over the entire band. In contrast, for large α, the subcarriers are more likely to be assigned to the Tx antenna with larger channel gains, which results in the increase of the achievable rate. Specifically, for $\alpha =\frac{64}{256}$, the proposed subcarrier allocation exhibits a slight performance degradation in MSE performance, but an approximately 16% increase in the achievable rate is achieved compared to the equi-space allocation method. Accordingly, by adjusting α, the radar/communication performances can be properly balanced.

**Figure 7:**
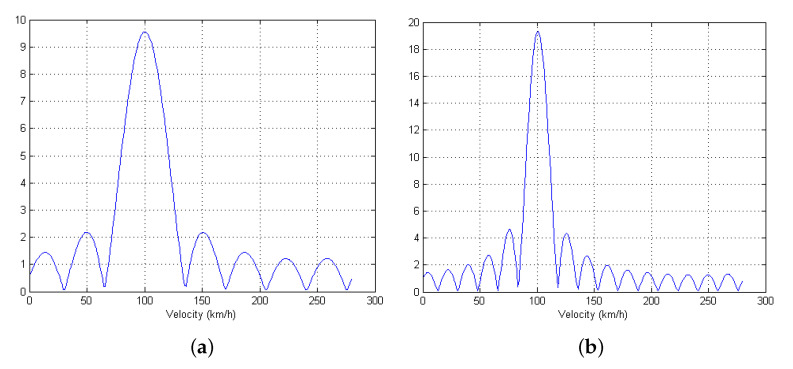
Doppler frequency (velocity) profile when Doppler estimation method in Section 6 is used with (**a**) $N_{sym}=8$ and (**b**) $N_{sym}=16$.

To verify the Doppler estimation method discussed in Section 6, when the radial velocity of a target is set as 100 km/h, the Doppler frequency profile ($F_{N_{sym}}{\bar{y}}_{doppler,j}$ in (28)) is displayed in Figure 7 for $N_{sym}=\left\{8,16\right\}$. From the figure, it can be found that the peak position coincides with the radial velocity of the associated target, and the resolution can be improved as the number of pulses increases, which is also induced from (28).

## 8. Conclusions

In this paper, by considering the RadCom platform equipped with multiple antennas using OFDM waveform, we propose the compressive sensing-based radar imaging and subcarrier allocation methods. Specifically, by exploiting the BMP-based imaging algorithm, 2D radar images with high resolution can be obtained, and by analyzing the effects of the subcarrier allocation on the achievable rate at the information and the MSE performance of our radar imaging problem, we also propose a new subcarrier allocation strategy that efficiently maximizes the achievable rate and simultaneously reduces the MSE of the radar images. From computer simulations, the BMP-based imaging method exhibits radar images with higher resolution, compared to the conventional backprojection-based imaging method. In addition, it is also verified that through the proposed subcarrier allocation strategy, a high achievable rate is obtained without sacrificing the radar imaging performance. In addition, by adjusting the subcarrier sub-block size, the radar/communication performances can be properly balanced.

## Figures and Tables

**Table 1:** $CRLB_{z}$ for various subcarrier allocation methods.

	${\mathrm{CRB}}_{z}$
**Equi-space**	74.6493
**Block-wise**	227.1595
**Pseudo-random**	75.3108

## Data Availability

The data presented in this study are available on request from the corresponding author. The data are not publicly available due to confidentiality reasons.
